# Supplementary material for: Anti-psychotic therapeutics modify gut microbial metabolism and modulate susceptibility to gastrointestinal infection in mice
Source: bioRxiv. 2026 Sep 16:2026.09.15.745804. Preprint. [Version 1] doi: 10.64898/2026.09.15.745804 (PMC13596375; doi:10.64898/2026.09.15.745804)
Supplement: Supplementary file 1 [file media-1.pdf]

Anti-psychotic therapeutics modify gut-microbial populations and modulate susceptibility to gastrointestinal infection in mice.

SUPPLEMENTARY DATA

C. Anthony Gacasan[1], Jaclyn Weinberg[2], Lyndsey D. Lipson[3], Gabrielle Webster[1], Dean P. Jones[2], Timothy Sampson[3], Rheinallt Jones[1], Michael H. Woodworth[4*]

Affiliations:

[1]Division of Gastroenterology, Hepatology and Nutrition Department of Pediatrics, Emory University School of Medicine, Atlanta, GA, USA.

[2]Division of Pulmonary, Allergy, Critical Care and Sleep Medicine, Department of Medicine, Emory University School of Medicine, Atlanta, GA

[3]Department of Cell Biology, Emory University School of Medicine, Atlanta, GA 30322, United States

[4]Division of Infectious Disease, Department of Medicine, Emory University School of Medicine, Atlanta, GA, USA

*Corresponding Author (michael.holmes.woodworth@emory.edu)

Key words: Anti-Psychotics, Microbiome

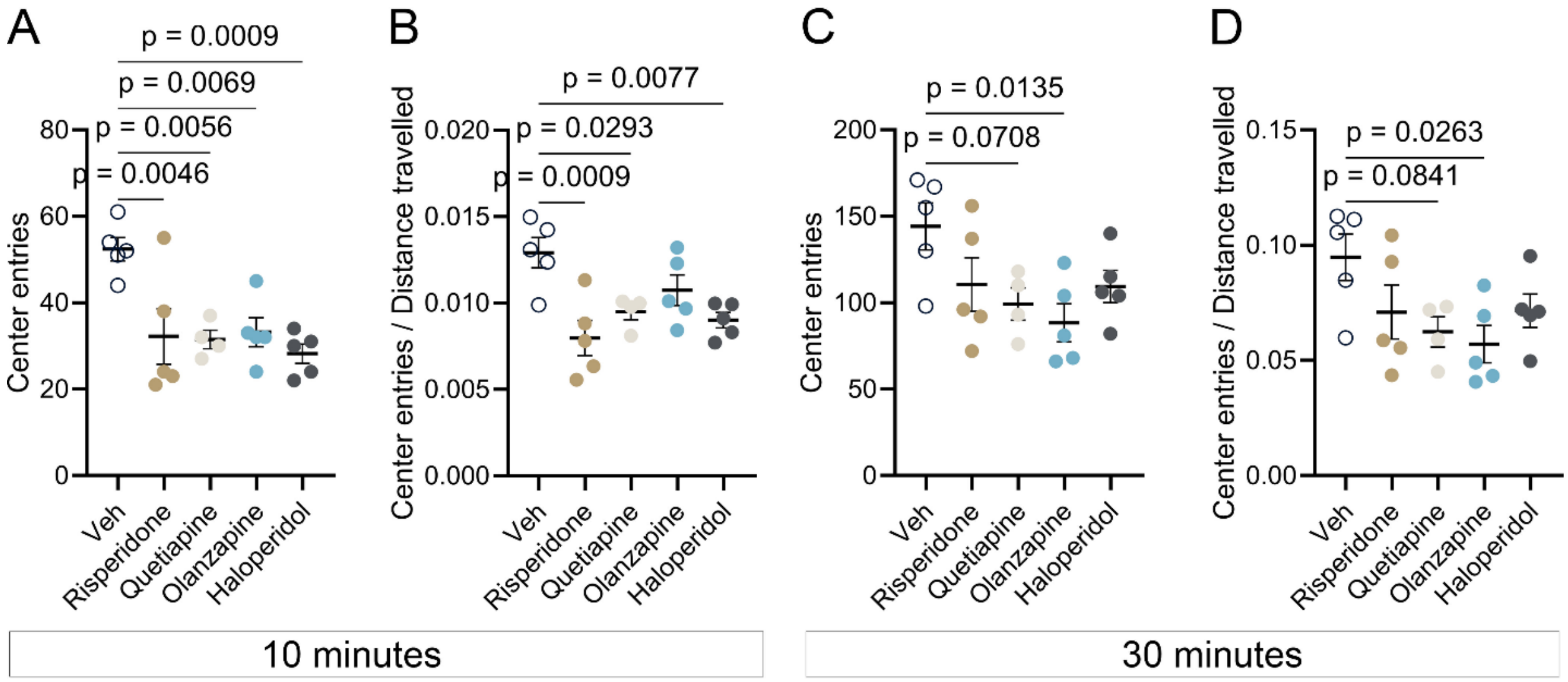

Supplementary Figure 1. Center-entry behavior during the open-field test. (A) Number of center entries during the first 10 minutes of the open-field test. (B) Center entries during the first 10 minutes normalized to distance traveled. (C) Number of center entries during the full 30-minute observation period. (D) Center entries during the 30-minute observation period normalized to distance traveled. Data are presented as mean ± SEM. P values were calculated using one-way ANOVA.

contrast	estimate	Standard Error	Degrees of Freedom	t-Statistic	p-value	Adjusted p-value (FDR)
Risperidone - Vehicle	0.26297	0.06952	62	3.782649	0.000352	0.001407
Olanzapine - Vehicle	0.142311	0.071972	62	1.977294	0.052459	0.157377
Haloperidol - Vehicle	0.101943	0.06952	62	1.466388	0.147597	0.295193
Quetiapine - Vehicle	0.023315	0.071145	62	0.327706	0.744238	0.744238

Supplementary Table 1. ANCOVA analysis of *C. rodentium* colonization dynamics over time. Log_{10}-transformed stool bacterial burden (CFU per gram of stool) was modeled as a function of time and treatment group using analysis of covariance (ANCOVA) to assess differences in temporal slopes between antipsychotic treatment groups and vehicle control. Reported statistics summarize treatment-specific slope estimates and associated significance testing, with multiple-comparison adjustment applied where indicated.

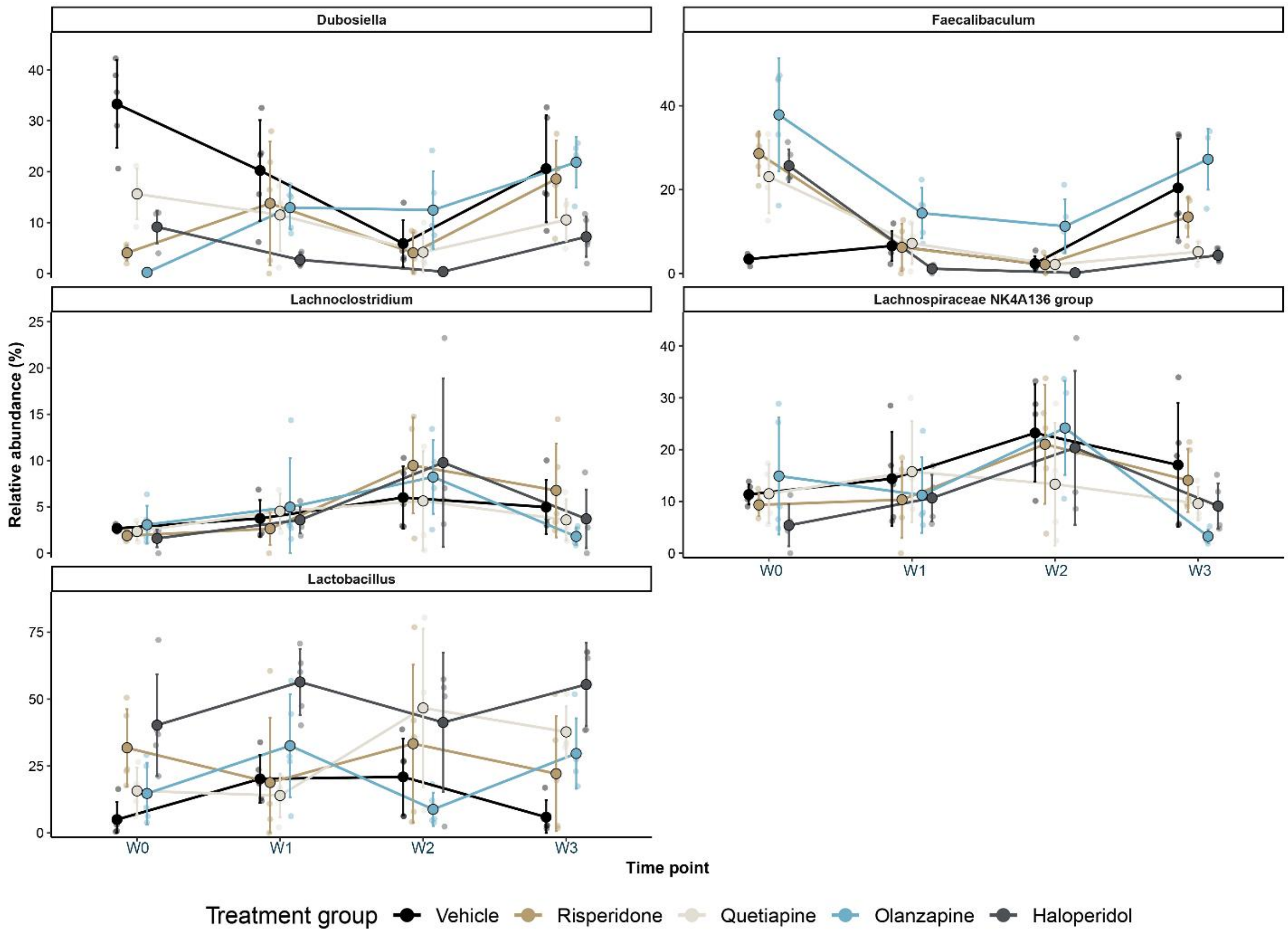

Supplementary Figure 2. Longitudinal changes in the relative abundance of dominant bacterial genera following antipsychotic treatment. Relative abundances of Faecalibaculum, Dubosiella, Lachnospiraceae NK4A136 group, Lachnoclostridium, and Lactobacillus are shown across W0, W1, W2 and W3 for vehicle-, risperidone-, quetiapine-, olanzapine-, and haloperidol-treated mice. Small points represent individual mice, large points represent group means, and error bars indicate mean ± standard deviation. Lines connect group means across time and are provided to visualize treatment-specific temporal patterns. Temporal trajectories were compared using generalized estimating equations with categorical time, treatment group, and their interaction, with mouse specified as the repeated-measures cluster and an AR(1) working correlation structure. Relative abundances were arcsine-square-root transformed before statistical analysis. Pairwise whole-trajectory Wald tests jointly compared between-group changes from baseline at (W0) at W1,

W2 and W3, with P values corrected within each taxon using the Benjamini–Hochberg false discovery rate procedure. Complete pairwise statistical comparisons are provided in Supplementary Table X.

Taxon	Group_comparison	df1	df2	F.ratio	p.value	Wald_chisq	Wald_df	p_FDR	Significance
Faecalibaculum	*Vehicle - Risperidone*	*3*	*75*	*44.073*	*1.58E-16*	*132.219*	*3*	*7.89E-16*	****
Faecalibaculum	*Vehicle - Quetiapine*	*3*	*75*	*33.989*	*5.58E-14*	*101.967*	*3*	*1.86E-13*	****
Faecalibaculum	*Vehicle - Olanzapine*	*3*	*75*	*17.905*	*7.33E-09*	*53.715*	*3*	*1.83E-08*	****
Faecalibaculum	*Vehicle - Haloperidol*	*3*	*75*	*69.542*	*1.31E-21*	*208.626*	*3*	*1.31E-20*	****
Faecalibaculum	*Risperidone - Quetiapine*	*3*	*75*	*2.689*	*0.052333*	*8.067*	*3*	*0.073574*	*ns*
Faecalibaculum	*Risperidone - Olanzapine*	*3*	*75*	*0.806*	*0.494417*	*2.418*	*3*	*0.494417*	*ns*
Faecalibaculum	*Risperidone - Haloperidol*	*3*	*75*	*8.498*	*6.26E-05*	*25.494*	*3*	*1.25E-04*	***
Faecalibaculum	*Quetiapine - Olanzapine*	*3*	*75*	*1.925*	*0.132834*	*5.775*	*3*	*0.147593*	*ns*
Faecalibaculum	*Quetiapine - Haloperidol*	*3*	*75*	*3.367*	*0.022935*	*10.101*	*3*	*0.038226*	*
Faecalibaculum	*Olanzapine - Haloperidol*	*3*	*75*	*2.593*	*0.058859*	*7.779*	*3*	*0.073574*	*ns*
Dubosiella	*Vehicle - Risperidone*	*3*	*75*	*14.98*	*9.85E-08*	*44.94*	*3*	*1.64E-07*	****
Dubosiella	*Vehicle - Quetiapine*	*3*	*75*	*3.45*	*0.020718*	*10.35*	*3*	*0.02302*	*
Dubosiella	*Vehicle - Olanzapine*	*3*	*75*	*35.16*	*2.69E-14*	*105.48*	*3*	*6.73E-14*	****
Dubosiella	*Vehicle - Haloperidol*	*3*	*75*	*4.878*	*0.003748*	*14.634*	*3*	*0.004685*	**
Dubosiella	*Risperidone - Quetiapine*	*3*	*75*	*35.434*	*2.27E-14*	*106.302*	*3*	*6.73E-14*	****
Dubosiella	*Risperidone - Olanzapine*	*3*	*75*	*18.607*	*4.03E-09*	*55.821*	*3*	*8.07E-09*	****
Dubosiella	*Risperidone - Haloperidol*	*3*	*75*	*13.825*	*2.89E-07*	*41.475*	*3*	*4.12E-07*	****
Dubosiella	*Quetiapine - Olanzapine*	*3*	*75*	*67.093*	*3.48E-21*	*201.279*	*3*	*3.48E-20*	****
Dubosiella	*Quetiapine - Haloperidol*	*3*	*75*	*0.447*	*0.720114*	*1.341*	*3*	*0.720114*	*ns*
Dubosiella	*Olanzapine - Haloperidol*	*3*	*75*	*50.373*	*6.11E-18*	*151.119*	*3*	*3.06E-17*	****
Lachnospiraceae NK4A136 group	*Vehicle - Risperidone*	*3*	*75*	*0.316*	*0.813969*	*0.948*	*3*	*0.813969*	*ns*
Lachnospiraceae NK4A136 group	*Vehicle - Quetiapine*	*3*	*75*	*2.506*	*0.065415*	*7.518*	*3*	*0.108321*	*ns*
Lachnospiraceae NK4A136 group	*Vehicle - Olanzapine*	*3*	*75*	*3.709*	*0.015147*	*11.127*	*3*	*0.050491*	*ns*
Lachnospiraceae NK4A136 group	*Vehicle - Haloperidol*	*3*	*75*	*0.882*	*0.454399*	*2.646*	*3*	*0.504887*	*ns*
Lachnospiraceae NK4A136 group	*Risperidone - Quetiapine*	*3*	*75*	*3.208*	*0.027827*	*9.624*	*3*	*0.069567*	*ns*
Lachnospiraceae NK4A136 group	*Risperidone - Olanzapine*	*3*	*75*	*14.924*	*1.04E-07*	*44.772*	*3*	*1.04E-06*	****
Lachnospiraceae NK4A136 group	*Risperidone - Haloperidol*	*3*	*75*	*2.385*	*0.075825*	*7.155*	*3*	*0.108321*	*ns*
Lachnospiraceae NK4A136 group	*Quetiapine - Olanzapine*	*3*	*75*	*2.938*	*0.038642*	*8.814*	*3*	*0.077285*	*ns*
Lachnospiraceae NK4A136 group	*Quetiapine - Haloperidol*	*3*	*75*	*2.073*	*0.110874*	*6.219*	*3*	*0.138593*	*ns*
Lachnospiraceae NK4A136 group	*Olanzapine - Haloperidol*	*3*	*75*	*6.199*	*8.07E-04*	*18.597*	*3*	*0.004033*	**
Lachnoclostridium	*Vehicle - Risperidone*	*3*	*75*	*2.277*	*0.086498*	*6.831*	*3*	*0.172996*	*ns*
Lachnoclostridium	*Vehicle - Quetiapine*	*3*	*75*	*1.053*	*0.374408*	*3.159*	*3*	*0.451601*	*ns*
Lachnoclostridium	*Vehicle - Olanzapine*	*3*	*75*	*4.404*	*0.006582*	*13.212*	*3*	*0.032909*	*
Lachnoclostridium	*Vehicle - Haloperidol*	*3*	*75*	*1.659*	*0.18303*	*4.977*	*3*	*0.305051*	*ns*
Lachnoclostridium	*Risperidone - Quetiapine*	*3*	*75*	*3.078*	*0.032567*	*9.234*	*3*	*0.108556*	*ns*
Lachnoclostridium	*Risperidone - Olanzapine*	*3*	*75*	*7.083*	*2.96E-04*	*21.249*	*3*	*0.002964*	**

Lachnoclostridium	*Risperidone - Haloperidol*	*3*	*75*	*2.658*	*0.054356*	*7.974*	*3*	*0.13589*	*ns*
Lachnoclostridium	*Quetiapine - Olanzapine*	*3*	*75*	*0.888*	*0.451601*	*2.664*	*3*	*0.451601*	*ns*
Lachnoclostridium	*Quetiapine - Haloperidol*	*3*	*75*	*0.955*	*0.418469*	*2.865*	*3*	*0.451601*	*ns*
Lachnoclostridium	*Olanzapine - Haloperidol*	*3*	*75*	*0.914*	*0.438609*	*2.742*	*3*	*0.451601*	*ns*
Lactobacillus	*Vehicle - Risperidone*	*3*	*75*	*4.387*	*0.006716*	*13.161*	*3*	*0.009594*	**
Lactobacillus	*Vehicle - Quetiapine*	*3*	*75*	*38.991*	*2.71E-15*	*116.973*	*3*	*2.71E-14*	****
Lactobacillus	*Vehicle - Olanzapine*	*3*	*75*	*8.82*	*4.42E-05*	*26.46*	*3*	*1.47E-04*	***
Lactobacillus	*Vehicle - Haloperidol*	*3*	*75*	*5.2*	*0.002566*	*15.6*	*3*	*0.004277*	**
Lactobacillus	*Risperidone - Quetiapine*	*3*	*75*	*10.648*	*6.54E-06*	*31.944*	*3*	*3.27E-05*	****
Lactobacillus	*Risperidone - Olanzapine*	*3*	*75*	*6.698*	*4.57E-04*	*20.094*	*3*	*0.001143*	**
Lactobacillus	*Risperidone - Haloperidol*	*3*	*75*	*2.648*	*0.055036*	*7.944*	*3*	*0.061151*	*ns*
Lactobacillus	*Quetiapine - Olanzapine*	*3*	*75*	*6.093*	*9.10E-04*	*18.279*	*3*	*0.001821*	**
Lactobacillus	*Quetiapine - Haloperidol*	*3*	*75*	*3.128*	*0.030657*	*9.384*	*3*	*0.038321*	*
Lactobacillus	*Olanzapine - Haloperidol*	*3*	*75*	*0.108*	*0.955236*	*0.324*	*3*	*0.955236*	*ns*

Supplementary Table 2. Pairwise comparisons of longitudinal relative-abundance trajectories among treatment groups for selected bacterial genera. Temporal trajectories of *Faecalibaculum*, *Dubosiella*, Lachnospiraceae NK4A136 group, *Lachnoclostridium*, and *Lactobacillus* were compared using generalized estimating equations with treatment group, categorical time, and their interaction as fixed effects and mouse as the repeated-measures cluster. Relative abundances were arcsine-square-root transformed before analysis. Wald tests jointly evaluated between-group differences in changes from baseline (W0) at W1, W2, and W3. The Benjamini–Hochberg procedure was applied to the 10 pairwise comparisons within each genus. Wald χ^2 tests had 3 degrees of freedom. Statistical significance was defined as BH-FDR $q < 0.05$. *$q < 0.05$; **$q < 0.01$; ***$q < 0.001$; ****$q < 0.0001$; ns, not significant.

	F.Model	R2	p-value	Adjusted p-value (FDR)
H_Post vs O_Post	7.1388	0.50491	0.028	0.035
H_Post vs Q_Post	16.182	0.69804	0.007	0.026
H_Post vs R_Post	1.6586	0.17172	0.229	0.229
H_Post vs V_Post	17.376	0.68475	0.007	0.026
O_Post vs Q_Post	19.089	0.76085	0.025	0.035
O_Post vs R_Post	7.5195	0.51789	0.02	0.033333
O_Post vs V_Post	3.8043	0.35211	0.032	0.035556
Q_Post vs R_Post	8.0274	0.53418	0.012	0.026
Q_Post vs V_Post	34.829	0.83265	0.008	0.026
R_Post vs V_Post	14.923	0.651	0.013	0.026

Supplementary Table 3. Pairwise permutational multivariate analysis of variance (PERMANOVA) results for untargeted metabolomics profiles across post-treatment groups. PERMANOVA was performed using all measured metabolomic features to assess differences in overall metabolic composition between each pair of treatment groups at the post-treatment time point. Reported statistics include the pseudo-F statistic (F.Model), proportion of variance explained (R^2), nominal p-values (pval), and multiple-testing–adjusted p-values (p.adj). Adjusted p-values were calculated to account for multiple pairwise comparisons.

Name	*mz_rt*	*Haloperidol Average Intensity*	*Olanzapine Average Intensity*	*Quetiapine Average Intensity*	*Risperidone Average Intensity*	*Vehicle Average Intensity*	*p_adj_fdr*	*HMDB Class*	*HMDB Sub Class*
Nornicotine	149.107318_104.3185079	26104574.8	39864430	11310177	24966371	62027410.4	1.98E-07	Pyridines and derivatives	
Diethylpropion	206.1538863_98.83902698	934757	1815774	537459.3	890646.2	2903758.2	2.93E-07	Organo ocygen compounds	
N-Feruloylglycyl-L-phenylalanine	399.155949_79.73455007	1203151	1658022	30950782	1043441.6	1256608.4	2.93E-07	Carboxylic acids and derivatives	Amino acids, peptides, and analogues
Castamollissin	469.0994193_150.3065675	0	0	223207.5	0	0	2.93E-07	Organooxygen compounds	Carbohydrates and carbohydrate conjugates
Phenelzine	137.1072823_97.18838279	8731314.4	15285975	4160314	8300971	18648463.4	3.21E-07	Benzene and substituted derivatives	Not Available
Myricetin 33-digalactoside	643.1503175_45.22017116	7515757	19275838	5585051	7312142	25523894.8	5.09E-07	Flavonoids	Flavonoid glycosides
Agrocybenine	207.1492354_100.4989209	16713295.6	34227483	8898023	15640620	43263666.8	6.18E-07	Pyrrolopyridines	Not Available
Arecoline	156.101941_288.0266019	455927.6	1444199	787487.8	816838.4	1681520.8	8.47E-07	Alkaloids and derivatives (SuperClass)	Not Available
Crotamiton	204.1383012_94.52925532	572902.6	1071530	248828	532272	2061861	1.20E-06	Benzene and substituted derivatives	Anilides
Lidocaine	235.1803785_93.62902346	17994288.6	37363840	10450742	17988323	52902968.4	1.20E-06	Benzene and substituted derivatives	Xylenes
19-Hydroxy-PGE2	351.2163006_56.82984427	4654404	6358501	2930249	3785197.2	7731277.2	1.64E-06	Fatty Acyls	Eicosanoids
Petunidin 3-galactoside	480.1247261_74.51755109	0	0	337577.5	0	0	1.64E-06	Flavonoids	Flavonoid glycosides
Gomphrenin II	697.1855184_45.46218913	616455.2	1254885	403038.8	549719.2	1917658.8	1.64E-06	Organooxygen compounds	Carbohydrates and carbohydrate conjugates
(-)-3-(2-methyl-3-furyl)thio-2-butanone	185.0632845_116.5893885	1762651	2094326	748843.5	1411016.8	2588423.8	1.84E-06	Thioethers	Aryl thioethers
(EE)-Piperlonguminine	274.1436673_94.12987461	1193384.8	2528360	423960.5	976559.8	3728121	1.84E-06	Benzodioxoles	Not Available
(Cyclohexylmethyl)pyrazine	177.1385509_89.34431704	21041414	50973701	13362560	24156460	80332087.6	2.29E-06	Diazines	Pyrazines
L(-)-Nicotine pestanal	163.1229478_97.20241098	11617332.6	20794116	5673837	12049022	29247137.2	2.73E-06	Pyridines and derivatives	Pyrrolidinylpyridines
2-Methoxy-3-methyl-9H-carbazole	212.1070117_92.6543102	505652.2	887951.8	333583.5	525482	1287828.6	2.81E-06	Indoles and derivatives	Carbazoles
L-Phenylalanine	166.0861563_120.5481476	10288863.2	11997062	5547659	9265538	15445977	3.22E-06	Carboxylic acids and derivatives	Amino acids, peptides, and analogues
DG(18:3(6Z9Z12Z)22:6(4Z7Z10Z13Z16Z19Z)0:0)	663.4964569_45.74883329	464743	1192361	328251	478695.2	1580755.4	3.22E-06	Glycerolipids	Diradylglycerols
2367-Tetrahydro-7-methylcyclopentbazepin-8(1H)-one	164.106993_104.1648628	19434048.6	27230408	7568724	17033627	37974176.6	4.03E-06	Not Available	Not Available
Mexiletine	180.1382658_111.982739	944286.2	1629348	551408.5	819103.8	1815107	4.10E-06	Phenol ethers	Not Available
Gadoversetamide	663.1595836_45.42759137	533219.2	1296175	427680.5	558787.4	1693204.6	4.30E-06	Not Available	Not Available
5678-Tetrahydro-24-dimethylquinoline	162.1276513_87.40353705	1066207.4	1957429	615456.3	1228821.2	3044595	4.56E-06	Quinolines and derivatives	Hydroquinolines
PG(18:018:0)	779.5786924_45.04181284	641086.6	1616777	574249.5	699685.2	2416890.6	4.60E-06	Glycerophospholipids	Glycerophosphoglycerols

NO-Didesmethyltramadol	236.1643497_101.6198847	1532236.8	2272254	807111.5	1549279.2	3402785.6	5.10E-06	Benzene and substituted derivatives	Cyclohexylphenols
4alpha-Formyl-4beta-methyl-5alpha-cholesta-824-dien-3beta-ol	409.3458408_47.75421323	512499.4	64465	653962.3	674579.8	0	5.92E-06	Prenol lipids	Triterpenoids
Tocainide	193.1335221_111.8710709	177427471.6	3.03E+08	86962336	151571742	379300067.2	5.92E-06	Benzene and substituted derivatives	Xylenes
PC(20:5(5Z8Z11Z14Z17Z)24:1(15Z))	890.6642249_45.78636381	351826.6	657073.8	222051.8	311565.8	896298.8	6.66E-06	Glycerophospholipids	Glycerophosphocholines
4-Amino-2-methyl-1-naphthol	174.091306_99.655045	1161259.2	2234088	784642.8	1258786	2806615.2	6.68E-06		
Kynuramine	165.1022653_127.5526863	31566857.6	45237190	14751656	33624749	62979039.8	7.53E-06	Organooxygen compounds	Carbonyl compounds
Cholesteryl acetate	429.3726654_47.83884678	32296665.2	749692.3	39444760	36352145	42914.8	8.24E-06	Steroids and steroid derivatives	Steroid esters
Tetrahydroharmol	203.1179045_116.1418549	5521338	8325396	2499932	5119269.2	11084577.6	8.24E-06	Harmala alkaloids	Not Available
N-Cyclopropyl-trans-2-cis-6-nonadienamide	176.1432981_80.76500173	1090134.2	1921665	523498.8	1561413.6	3849442.6	1.16E-05	Fatty Acyls	Fatty amides
1-Cyano-2-hydroxy-3-butene	98.06005797_36.87901628	173814.4	282871.3	224931.8	187072.8	282022.8	1.38E-05	Organooxygen compounds	Alcohols and polyols
lysoPC(26:1(5Z))	634.4781275_46.36550682	258989.6	562329.8	147100.5	238720.6	738681.8	1.72E-05	Glycerophospholipids	Glycerophosphocholines
Dictyoquinazol A	313.1192955_104.7398639	885889.6	804533.3	4859186	713113	875143	1.72E-05	Diazanaphthalenes	Benzodiazines
2-hydroxymexiletine	196.1332089_105.4920339	13250047.2	20776773	6640363	11378178	23769388.6	1.72E-05	Phenols	4-alkoxyphenols
Metanephrine	198.1124197_143.5468325	6471391	8093136	2970160	5442955.8	8546176.4	1.74E-05	Phenols	Methoxyphenols
91013-Trihydroxystearic acid	333.2635902_57.75068644	12926192.4	15357533	4692815	7843860.6	19592390.2	1.77E-05	Fatty Acyls	Fatty acids and conjugates
Hexylcaine	262.1799698_90.46435654	1214866	1308012	467853	1313789.4	2776608.4	1.81E-05	Benzene and substituted derivatives	Benzoic acids and derivatives
Cyclopentolate	292.1906435_91.73460507	1123363.2	1426687	466662	1167837	2215912.2	2.10E-05	Benzene and substituted derivatives	Not Available
Spinosin C	755.2180662_45.96330447	354906.4	781138.3	315200.8	346776.6	1166125	2.18E-05	Flavonoids	Flavonoid glycosides
1-(23-Dihydro-1H-pyrrolizin-5-yl)-14-pentanedione	206.1175322_102.5465388	12080714	16500091	5942034	13453923	26867961.2	2.22E-05	Carboxylic acids and derivatives	Amino acids, peptides, and analogues
Nizatidine	332.1225878_3.746346135	0	0	122641.9	0	0	2.26E-05	Azoles	Thiazoles
Mepivacaine	247.180474_90.77703602	2590125.8	5316368	1684918	2966797	7463562.8	2.26E-05	Piperidines	Piperidinecarboxylic acids and derivatives
S-Ethyl thioacetate	105.0368462_174.2860294	404522.8	808212.3	198007.5	522394.2	1260368	2.30E-05	Thiocarboxylic acids and derivatives	Thioesters
5678-Tetrahydroquinoxaline	135.0916016_117.651223	34163326.2	45819091	15141099	30633181	58161786.8	2.30E-05	Diazines	Pyrazines
5-Methoxydimethyltryptamine	219.1491763_96.41654362	7790326.6	13380669	4401435	8241204.6	19180730	3.05E-05	Indoles and derivatives	Tryptamines and derivatives
Prostaglandin E2	353.2319464_57.20320237	5194545	6757572	2787330	3880331.4	8136562.2	3.69E-05	Fatty Acyls	Eicosanoids

Supplementary Table 4. Top 50 putatively annotated metabolites identified from untargeted metabolomics analysis using xMSannotator. Metabolites are ranked by false discovery rate–adjusted p-values (p_adj_fdr) derived from differential abundance testing across antipsychotic treatment groups and vehicle. For each feature, the table reports the putative compound name, m/z–retention time identifier (mz_rt), average feature intensity within each treatment group, FDR-adjusted p-value, and Human Metabolome Database (HMDB) chemical class and subclass where available. Annotations are putative and based on accurate mass, retention time, and database-supported chemical inference.

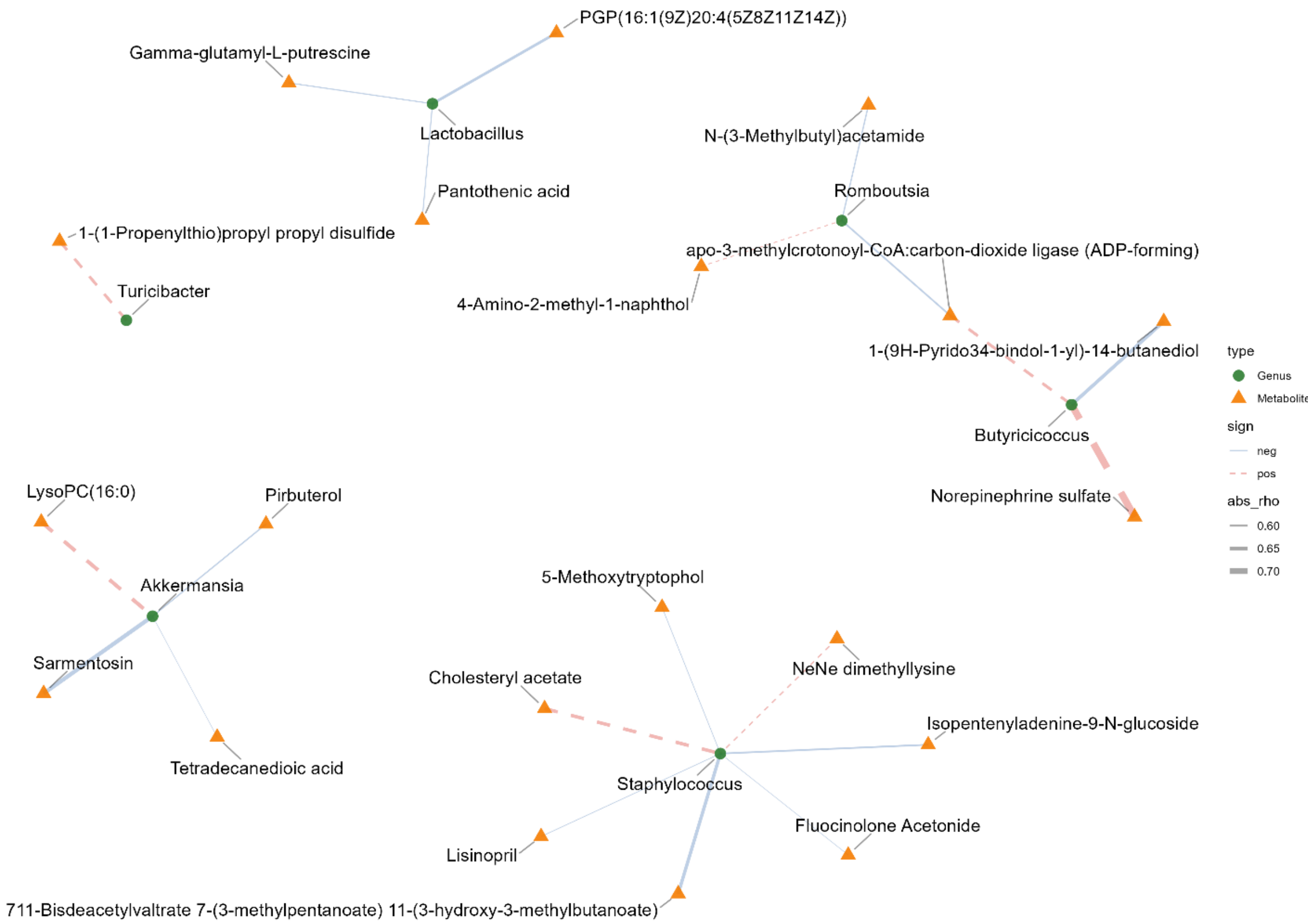

Supplementary Figure 3. Selected hub genera from the bipartite genus–metabolite association network constructed using the strongest correlations for visualization. Nodes represent genera (green circles) and metabolites (orange triangles). Edges are weighted by the absolute value of the correlation coefficient (|ρ|; thicker edges indicate stronger associations) and are colored and styled by directionality (positive = red dashed; negative = blue solid). Highlighted genera include *Akkermansia*, *Lactobacillus*, *Turicibacter*, *Butyricicoccus*, *Staphylococcus*, and *Romboutsia*, along with their first-degree (1-hop) neighboring metabolites.

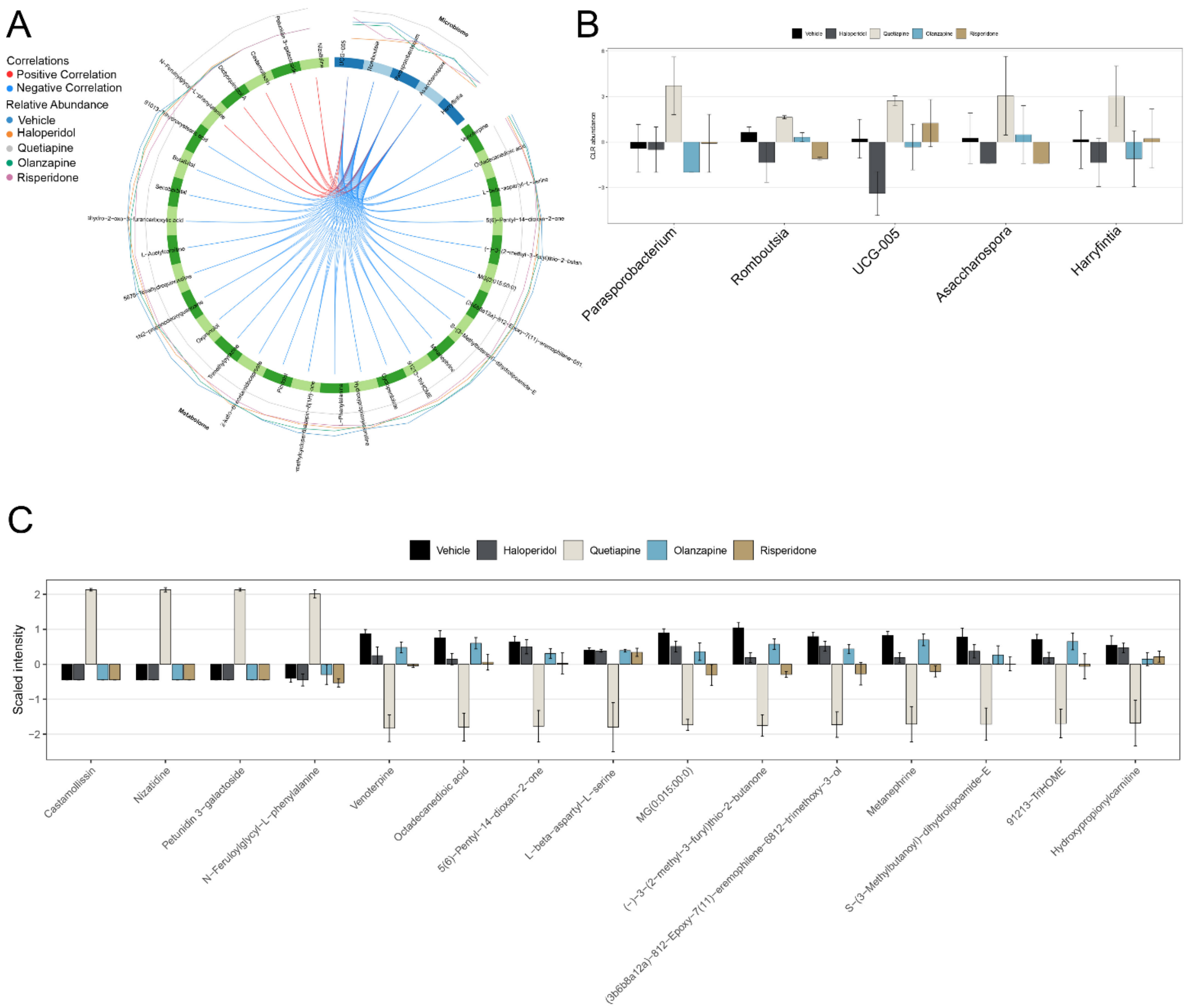

Supplementary Figure 4. DIABLO integration of microbiome and metabolome features at week 3. (A) Circos plot showing genus-level microbial features and putatively annotated metabolites selected by the DIABLO block.sPLS-DA model at T6. Outer tracks represent the relative contribution of each selected feature to treatment-group discrimination, and connecting lines indicate cross-omics correlations on component 1 with $|r| \geq 0.4$. Red lines indicate positive correlations and blue lines indicate negative correlations. **(B)** CLR-transformed abundances of the selected bacterial genera across treatment groups. **(C)** Scaled intensities of the selected putatively

annotated metabolite features across treatment groups. Bars represent group means with error bars indicating SEM. Microbiome data were CLR-transformed following prevalence and variance filtering. Metabolomics data were log2-transformed, imputed using half of the minimum detected value, and variance-filtered before integration.
